# The complete chloroplast genome of *Chrysanthemum boreale* (Asteraceae)

**DOI:** 10.1080/23802359.2018.1468225

**Published:** 2018-04-28

**Authors:** So Youn Won, Jae-A Jung, Jung Sun Kim

**Affiliations:** a Genomics Division, National Institute of Agricultural Sciences, Rural Development Administration, Jeonju, Republic of Korea;; b Floriculture Research Division, National Institute of Horticultural and Herbal Science, Rural Development Administration, Wanju, Republic of Korea

**Keywords:** Chrysanthemum, chloroplast genome, PacBio SMRT sequencing, Asteraceae

## Abstract

*Chrysanthemum boreale* is a perennial plant in the Asteraceae family that is native to eastern Asia and has both ornamental and herbal uses. Here, we determined the complete chloroplast genome sequence for *C. boreale* using long-read sequencing. The chloroplast genome was 151,012 bp and consisted of a large single copy (LSC) region (82,817 bp), a small single copy (SSC) region (18,281 bp) and two inverted repeats (IRs) (24,957 bp). It was predicted to contain 131 genes, including 87 protein-coding genes, eight rRNAs and 46 tRNAs. Phylogenetic analysis of chloroplast genomes clustered *C. boreale* with other *Chrysanthemum* and Asteraceae species.

Members of the *Chrysanthemum* genus are used around the world as ornamental, herbal and medicinal plants and belong to Asteraceae, one of the largest angiosperm families. *Chrysanthemum boreale* is a wild species native to eastern Asia (Korea, China, and Japan) and diverged from the commercial cultivar, *Chrysanthemum morifolium*, 1.7 million years ago (Won et al. 2017). As *C. boreale* exhibits strong resistance to white rust disease caused by *Puccinia horiana*, it has been suggested as a genetic resource in chrysanthemum breeding to develop disease-resistant cultivars (Park et al. 2014). Due to its yellow floret, however, *C. boreale* is sometimes difficult to distinguish from other yellow chrysanthemums, such as *Chrysanthemum indicum*. As such, obtaining the complete chloroplast sequence will enable identification by molecular markers.

In this study, we reconstructed the chloroplast genome of *C. boreale* (NCBI BioSample SAMN07296937) using long-read sequencing data generated by PacBio’s Single Molecule Real-Time (SMRT) system, in which the average read length was between 10 and 15 kb. The plant of *C. boreale* was collected from Jangseong-gun, Jellanam-do, Republic of Korea (GPS location N 35° 29′ 00″ E 126° 48′ 00″) and was maintained in the National Institute of Horticultural and Herbal Science, Rural Development Administration under the identification number of IT121002 (Hwang et al. 2013). The sequencing library was prepared using total genomic DNA and SMRTbell Template Prep Kit 1.0 (Pacific Biosciences, PN 100-259-100) and sequenced on PacBio’s RS II platform with P6-C4 chemistry by DNAlink (Republic of Korea). After the genome was initially assembled using the FALCON and FALCON-Unzip algorithms (Chin et al. 2016), chloroplast-like reads were isolated by conducting BLASTn analysis against the complete chloroplast genome of *Chrysanthemum indicum* (NCBI Accession number: JN867592). The obtained reads were further assembled using CANU (Version 1.4) with the genome size set at 400,000 (Koren et al. 2017). Finally, the assembly was circularized by comparing the contig end sequences by MUMmer (Kurtz et al. 2004). The chloroplast genome was annotated using the Dual Organellar GenoMe Annotator (DOGMA) program (Wyman et al. 2004) and deposited into GenBank under accession number MG913594.

The *C. boreale* chloroplast genome was 151,012 bp in length with an overall GC content of 37.47%. It exhibited a canonical quadripartite structure with an large single copy (LSC) region of 82,817 bp, an small single copy (SSC) region of 18,281 bp and a pair of 24,957 bp inverted repeats (IRs). Genome annotation revealed 131 functional genes including 87 protein-coding genes, eight rRNAs, and 36 tRNAs. Among the genes, 17 contained one intron, two included two introns, and one was trans-spliced. Genes located in the IR regions, including four rRNAs were found to be duplicated, which was commonly observed in other Asteraceae species (Zhang et al. 2016).

A phylogenetic analysis was performed using the complete chloroplast genome of *C. boreale* with those from five Asteraceae species, including two from the genus *Chrysanthemum* and three from another genus. *Nicotiana tabacum* (Solanaceae) was used as an outgroup. The maximum likelihood-based analysis showed that *C. boreale* was clustered with other *Chrysanthemum* species, with high bootstrap values (Figure 1). The phylogenetic relationships of the analysed Asteraceae species were consistent with previous results (Panero and Crozier 2016). The chloroplast sequence of *C. boreale* can be used for species identification and phylogenetic analysis.

**Figure 1.**
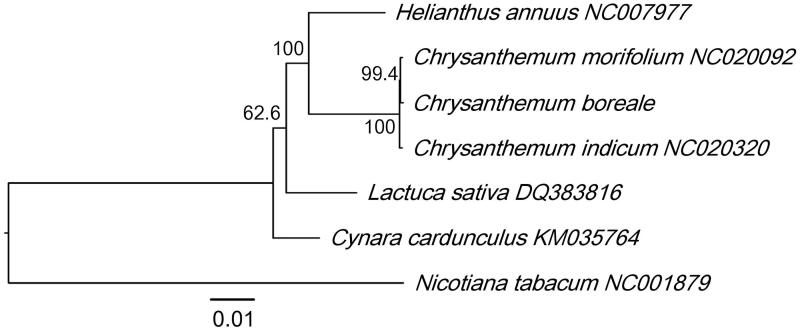
Maximum-likelihood phylogenetic tree based on complete chloroplast genome sequences of *C. boreale*, five other Asteraceae species, and *N. tabacum*. The numbers on the branches indicate bootstrap support values from 1000 iterations. Scale bar is substitutions per site.
